# LeMoRe-MTKD: A Multi-Teacher–Student Knowledge Distillation Deep Learning Framework for Landslide Segmentation Using UAV Imagery

**DOI:** 10.3390/s26216750

**Published:** 2026-10-25

**Authors:** Seunghyo Ahn, Nguyen Hong Quang, Geonhwi Jung, Bomi Kim, Joowon Park

**Affiliations:** 1 Department of Smart Infrastructure Disaster Prevention, Kangwon National University, 7 Jukheon-Gil, Gangneung-si 25457, Republic of Korea; sha@kangwon.ac.kr; 2 Institute for Smart Infrastructure, Kangwon National University, 7 Jukheon-Gil, Gangneung-si 25457, Republic of Korea; r01146@kangwon.ac.kr; 3 Faculty of Multimedia, Posts and Telecommunications Institute of Technology, Hanoi 11307, Vietnam; 4 Department of Forestry, Kyungpook National University, 80 Daehak-Ro, Buk-Gu, Daegu 41566, Republic of Korea; alzaki3050@knu.ac.kr; 5 Forest Ecosystem Restoration Division, Korea Forest Service, 189 Cheongsa-Ro, Seo-Gu, Daejeon 35208, Republic of Korea; bm0901@korea.kr; 6 School of Forest Science and Landscape Architecture, Kyungpook National University, 80 Daehak-Ro, Buk-Gu, Daegu 41566, Republic of Korea

**Keywords:** knowledge distillation, rainfall-triggered landslides, South Korea, UAV imaging, weight tuning

## Abstract

Landslides (LSs) are a natural phenomenon, typically triggered by heavy rainfall and earthquakes, and pose a global danger to human lives and property. LSs frequently occur on steep slopes and in loose soil conditions, and damaged sites can be hard to reach thereafter. Thus, remote approaches would be relevant and practical for investigations, and the use of an Unmanned Aerial Vehicle (UAV) for imaging, along with robust methods to identify and quantify LS-affected areas, is recommended. Therefore, this study proposed a multi-teacher–student knowledge distillation (KD) deep learning framework for landslide semantic segmentation. The lightweight LeMoRe model is selected for the learner model (student), and SegNet, U-Net, DeepLabV3+, and LinkNet are used as the teacher networks. To establish the UAV training dataset, we conducted UAV imaging for four LS sites, including Mogo, Buri, Oejeong, and Sangneung (testing site) villages, in Sancheong County, South Korea, in 2025. First, all plain networks are trained on the training dataset, and then KD is applied for single-teacher and multi-teacher KD. Subsequently, ablation studies are implemented to identify the optimal KD component set and the best student–teacher combination (with equal weights) for subsequent experiments. In addition, the equal-weight combination is tuned for the optimal teacher weight proportion to maximize the teacher knowledge transfer to the student. The best model weight is saved for inference with the testing site of Sangneung to map the LS-damaged areas. The experiment results reveal an improvement in accuracy (6.3% F1, 8.2% IoU with equal weight and 12.3% F1 and around 16.8% IoU with the tuned weight). The inference results for the Sangneung testing site were qualitatively and quantitatively evaluated through visual comparison with the original UAV imagery with three zoom-in areas presented for detailed examination. Finally, efficiency, the trade-off between accuracy and efficiency, limitations, and practical KD are discussed.

## 1. Introduction

The natural process of downward movement of soils and rocks in mountainous areas is known as landslides (LSs), which pose critical challenges at the global scale and draw much attention from managers and the scientific community [1], as they can threaten human life, endanger infrastructure, and destroy the land [2]. Globally, LSs have resulted in 56 thousand fatalities and around USD 20 billion in annual economic loss between 2004 and 2016 [3]. The World Bank reported that 3.7 million km^2^ of land is highly prone to LSs, putting more than 300 million people in danger [4]. South Korea has environmental conditions where 79% of the nation’s territory is mountainous, with soil strength, slope stability, and heavy rains, which create favorable conditions for landslide occurrence [5]. The Korea Forest Service (KFS) reported that 13,676 ha of land, more than 300 casualties, and USD 1.5 billion were lost due to LSs over the 30-year period from 1993 to 2022 [6]. Most of the LS events correspond to typhoons carrying heavy rains, which trigger massive landslides in the summer season (July to September) [7,8]. The importance of the LS issue is indicated by the South Korean government and research institutions in their efforts to develop models for predicting and providing early warnings of landslides in order to minimize damage [9].

Remote sensing data are increasing in availability in spatial and temporal resolutions with both commercial and free-of-charge data, leading to a surge in their applications in general and for LS studies in particular [10]. Remote sensing techniques are also diverse in sensor technology, such as multispectral, hyperspectral, synthetic aperture radar (SAR), and orbit heights (spaceborne, airborne, UAV, and ground-based observations). The diversity of remote sensing data supports LS research with various applications, including investigations, prediction, mapping, and early warning for both slow and rapid LS studies [11,12]. In addition, remote sensing technology provides a wide range of data on land cover classes, digital elevation models, and precipitation data (from meteorological satellites), e.g., for LS-susceptibility research, which frequently involves data-driven studies including non-remote-sensing information [13,14,15]. Furthermore, unmanned aerial vehicle (UAV) technology has been developed to an advanced stage with higher-quality sensors and, combined with AI and edge computing techniques, the use of UAV data has high potential for LS research and applications.

Along with the advantages of UAV sensors, the challenges of LS segmentation using UAV data remain due to the high-density vegetative cover where LS can occur. With low-altitude flights, the effects of vegetation cover (cover and shade) and terrain distortion should be taken into account; small LSs can be hindered beneath them [16]. Thus, the accuracy of LS maps can be reduced despite the advanced AI technique applied. Even with centimeter-scale spatial resolution and more spectral bands being sensed with current modern sensors [17], UAV imaging is not as frequently observed as space-borne remote sensing, which is limited for monitoring applications. However, as UAV imaging is easy to conduct on site, it is extremely useful during and post-LS investigations, leading to a revolution in landslide investigation by surpassing the complexity of in situ surveys and being time- and cost-effective [18]; in some cases, it is impossible to survey cut-off areas due to severe LS damage [19]. In addition, except for the inclusion of geographical coordinates in UAV imagery, it is quite similar to ground-based imagery, as most deep learning models are trained using them. Thus, knowledge transfer is more convenient compared to satellite imagery acquired from distant space orbits. The development of applications for UAV imagery is partly due to its integration with modern AI technology, especially with convolutional neural network (CNN) structures. However, we may still be in the early stages of development, so there is still much work to be conducted with UAV data and CNN models.

Working with imagery, in the AI domain, deep learning (DL) models are frequently used for object detection and segmentation tasks. In LS recognition, DL models can be classified into two categories: object-based and pixel-based approaches [20]. In LS delineation, DL-based semantic segmentation has become a mainstream pixel-wise approach, where CNNs have been developed with high accuracy and efficiency. Examples of large CNNs are DeepLabV3 series [21], Segment Anything Model (SAM) [22], YOLO series with x-size encoder [23], moderate-size CNNs such as U-Net [24], PSPNet [25], LinkNet [26], and lightweight models such as LeMoRe [27], EfficientNet [28], and EfficientSegNet [29]. However, complex CNNs often require powerful computational resources but gain higher accuracy compared to lightweight networks. This is an inherent conflict that is challenging to solve. As the computational efficiency of lightweight CNNs has increased, attention has been paid to improving the drawback of lower accuracy and overfitting when training with small datasets by modifications or adding components to existing networks [30]. Another direction on lightweight CNNs is model stacking or ensembling that combines a series of small pretrained CNNs to gain higher accuracy without retraining the baseline architectures, based on determining the weights of the component models, such as [31,32,33,34]. However, this combination may face a limitation of dependence on the baseline models’ predictions in the inference phase, which may take more time compared to the time of inferencing a single model.

Hence, this study proposed a novel framework of knowledge distillation (KD) from heavy and moderate-sized CNNs (teachers) to the lightweight architecture (student) to achieve better accuracy from the student. Specifically, the LeMoRe (student) distills knowledge from teachers (SegNet, U-Net, DeepLabV3+, and LinkNet) to harness knowledge from the teacher models and pretrained backbones, and we expect the distilled model (student), called LeMoRe-TSKD, to have better performance for landslide semantic segmentation using UAV imagery. In addition, the model performances will be evaluated and compared with the original full distilled model and ablated versions. The results show the potential for improving the baseline LeMoRe through multi-teacher KD and achieving a higher IoU than LeMoRe-Plain and the single-teacher KD configurations. However, without a comparison against a more highly optimized baseline student or a direct teacher ensemble, the entire performance improvement solely by the KD mechanism has not been confirmed. There are four main contributions of this work:

(1) Conducting UAV scanning of four LS sites to obtain high-resolution orthophotos, and field investigating the LS boundaries, updating them in the room.
(2) Processing and establishing training/validating and testing data.
(3) Training the teacher models, student, and the LeMoRe-TSKD, and evaluating the DL model performance.
(4) Ablation study to find the best architecture for model inference and mapping the LS-damaged areas.

The rest of the paper is organized as follows: Section 2 and Section 3 present the related works and study areas, and the materials and methods are described in Section 4. The study results, discussion, and conclusion are presented in Section 5, Section 6 and Section 7, and the back matter is presented in the remaining parts, respectively.

## 2. Related Works

### 2.1. Deep Learning for Landslide Detection and Segmentation

Deep learning architectures have been used widely for landslide detection and segmentation with remote sensing and UAV data, as they can produce results promptly, such as [35], by enhancing YOLO with shallow detection heads. Amuthadevi et al. (2026) [36] integrated multi-temporal satellite and UAV images for LS detection and achieved an IoU of 0.89 with the Seg-Net model. In addition, Gui et al. (2026) [37] proposed a novel object-based residual graph attention architecture for LS in the Himalayan region, which achieved an F1-score of around 0.92. He et al. (2025) [38] deployed the latest YOLOv11-seg model for LS detection and segmentation in comparison with different-sized models of YOLOv11-seg. Furthermore, Attention Residual U-Net (ARU-Net) was employed in the baseline U-Net to improve model performance by [39] and accuracy improving by 5.13% and mIoU by 11.63% compared to a previous study’s results. Similarly, [40] integrates an Attention Selective Kernel (ASK) into the baseline U-Net for improving semantic segmentation. For wide study areas, Jichao et al. (2025) [41] developed a pixel-level contrastive learning semi-supervised semantic segmentation model for the aim of LS detection, achieving an F1-score of 0.91 and mIoU of 0.84. Lastly, a combination of semantic segmentation and susceptibility assessment was completed by Rezaie et al., 2025 [42] with the red, green, blue plus depth (RGB-D) fusion network (RDFNet), the CNN-whale optimization algorithm and CNN-Harris hawk optimization (HHO). The study found that RDFNet was the best model with the highest F1-score of 0.67, precision of 0.55, recall of 0.84, and accuracy of 0.92.

### 2.2. Lightweight Segmentation Models

As mentioned, lightweight CNNs have the advantage of consuming less powerful computational infrastructure and are typically fast. Thus, many attempts have deployed and developed novel light networks for both detection and segmentation purposes. Zhang et al. (2025) [43] proposed a new EoML-SlideNet, a lightweight architecture for LS displacement prediction for multiple monitoring data. The EoML-SlideNet shows two to four times lower MAE/RMSE than most popular DL models, and it provides 3–30 times faster inference. In addition, the LSMamba, an LS segmentation architecture that uses the VSSD backbone and removes the heavy convolutional fusion heads to achieve both accuracy improvement and reduced computational cost, is proposed by Tang et al. (2025) [44]. Most recently, to overcome the challenges of post-disaster investigation in a mountainous environment, Gu et al. (2026) proposed a “three-act on-board 3D modelling framework” for geological disaster assessment, including landslides, which is a modified version of YOLOv11-seg size nano (n) [45]. What is more, co-seismic landslides are identified by a lightweight semantic segmentation network, which is the “Stepwise Lightweight Adaptive Transfer Learning (SLATL) framework” aiming to balance accuracy and efficiency [30]. The model was validated using the Hokkaido and Wenchuan datasets and harnessed the mIoU of 0.83. Another method, “Adaptive Feature Extraction Lightweight Network for Landslide Detection (AFL-NET)”, is proposed by Pan and Pan (2026) for LS detection enhancement and claims an improvement in mAP of 3.9% and a 15.3% reduction in the model parameters [46].

### 2.3. Knowledge Distillation for Landslide Detection and Segmentation

Recently, there have been attempts to distill knowledge from source models (teacher) to target networks (student) with the aim of improving accuracy but without causing the student model to increase in size. This research direction is considered promising and has potential, while CNNs have been developed to advance. Huang and Chan (2024) [47] developed an innovative knowledge distillation CNN based on the Swin-Transformer named SwinLiteKD, which is aimed at post-disaster rescue operations. SwinLiteKD presented superior computational efficiency and achieved an overall accuracy of 0.97. Another method, “knowledge distillation adversarial (KDA)” is introduced by Wang et al. (2025) [48] for LS segmentation using remote sensing data. KDA leveraged the vision foundation models (VFMs) and the dynamic cross-layer fusion (DCF) decoder for global-local feature interaction enhancement. The experiment achieved a precision of 0.92 and an mIoU of 0.86. Tagud and Lumauag (2026) [49] enhanced LS detection by knowledge distillation and an ensemble of the baseline U-Net with multiple teachers. The experimental results show the optimal balance with the use of the Focal Loss in the Attention U-Net with the detection of 99.6% of correct landslide pixels. Additionally, self- and automated distillation based on the original DINO baseline is applied by [50,51] using high-resolution and multiple remote sensing data, presenting the robustness of DINOv2 compared with other popular models of SimCLR, SwAV, and ResUnet-50.

### 2.4. UAV-Based Remote Sensing for Disaster Studies

In terms of smaller study scales, the data (imagery) from unmanned aerial vehicles (UAVs) have been frequently used in the AI deep learning domain for disaster mapping, including landslide mapping (LSM) for several purposes: first, for LS recognition by identifying the LS boundaries and cracks on the ground surface. Cheng et al. (2025) fused the CNN’s ability to accurately segment with a deep transfer learning strategy for crack recognition on UAV images [52]. Li et al. (2024) [20] exploit DeepLabV3+ with “ the rationale behind the dual-encoder design” for model validation and the PE loss for the ablation study experiment. The model gained an mIoU of 76.0% and a 5.5% improvement in accuracy. Secondly, for LS deformation monitoring tasks, Jiang et al. (2022) fused the terrestrial laser scanning (TLS) with UAV photogrammetry and ground control points (GCPs) to reconstruct the UAV model for calculating the LS displacement [53]. In addition, a combination of UAV, UAV-Lidar, GNSS, and InSAR technologies is used for the LS monitoring task, such as [18,54,55,56,57], as these techniques produce surface elevation models that are useful for land surface deformation (movement) monitoring aims. Thirdly, for the LS mapping orientation, Șandric et al. (2024) employed U-Net and DeepLab CNN to segment LS-damaged areas in Romania and concluded that the U-Net was the winner; however, choosing the appropriate model should be based on specific requirements and prior accuracy metrics [17]. Finally, an example of LS detection using UAV imagery and a deep learning model is the recent study of Zhou et al. (2026), which proposed a so-called “wavelet-guided semantic decoupling for landslide detection (WSDLNet)”, which supposedly has the ability to discriminate the spectral and spatial domains and identify landslide areas under diverse geological conditions [58].

## 3. Study Areas

**Figure 1:**
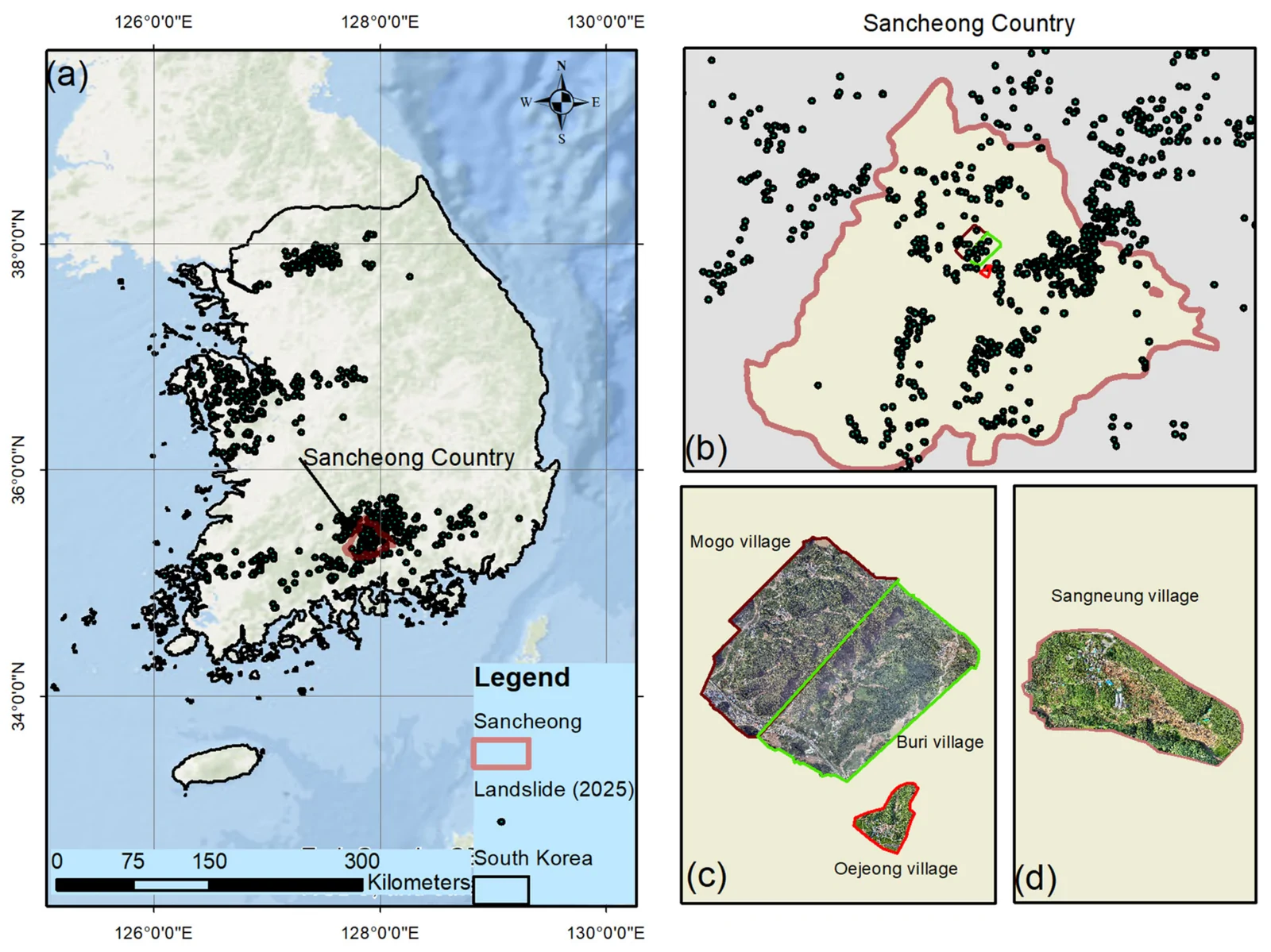
Landslide study areas and used UAV images; (**a**) location of Sancheong Country (in pink) in South Korea, (**b**) locations of landslides that occurred in 2025 (the circles), Mogo village (dark red), Buri village (light green), Oejeong village (red), and Sangneung village (pink), (**c**,**d**) are UAV images of the four villages.

The year 2025 was a severe year of landslides in South Korea, with more than 5 thousand (rainfall-triggered) LS affected locations distributed in the north, mid-west, and in the south (Figure 1a). The Sancheong County (district) Gyeongsangnam province, located at 35°15′–35°35′ N latitude and 127°40′–128°5′ E longitude, was among the most severely devastated district in the nation in the rainy season in 2025 (Figure 1b). The elevation of the district ranges from around 40 to 1920 m above the sea level and the terrain is characterized by a deep-incised drainage network, together with East Asian humid temperature climate and annual rainfall ranges from 1400 to 1800 mm [59], creating an LS-prone condition of the region. Due to the heavy rains from 16 to 19 July 2025, there were many landslide locations in Sancheong County; however, in this study, we used UAV images scanned a few days after the event of four villages: Mogo, Buri, and Oejeong (Figure 1c) and Sengneung village (Figure 1d) as part of the county.

## 4. Materials and Methods

### 4.1. UAV Imaging, In-Room Data Processing, and Ground-Truth LS Masking

**Figure 2:**
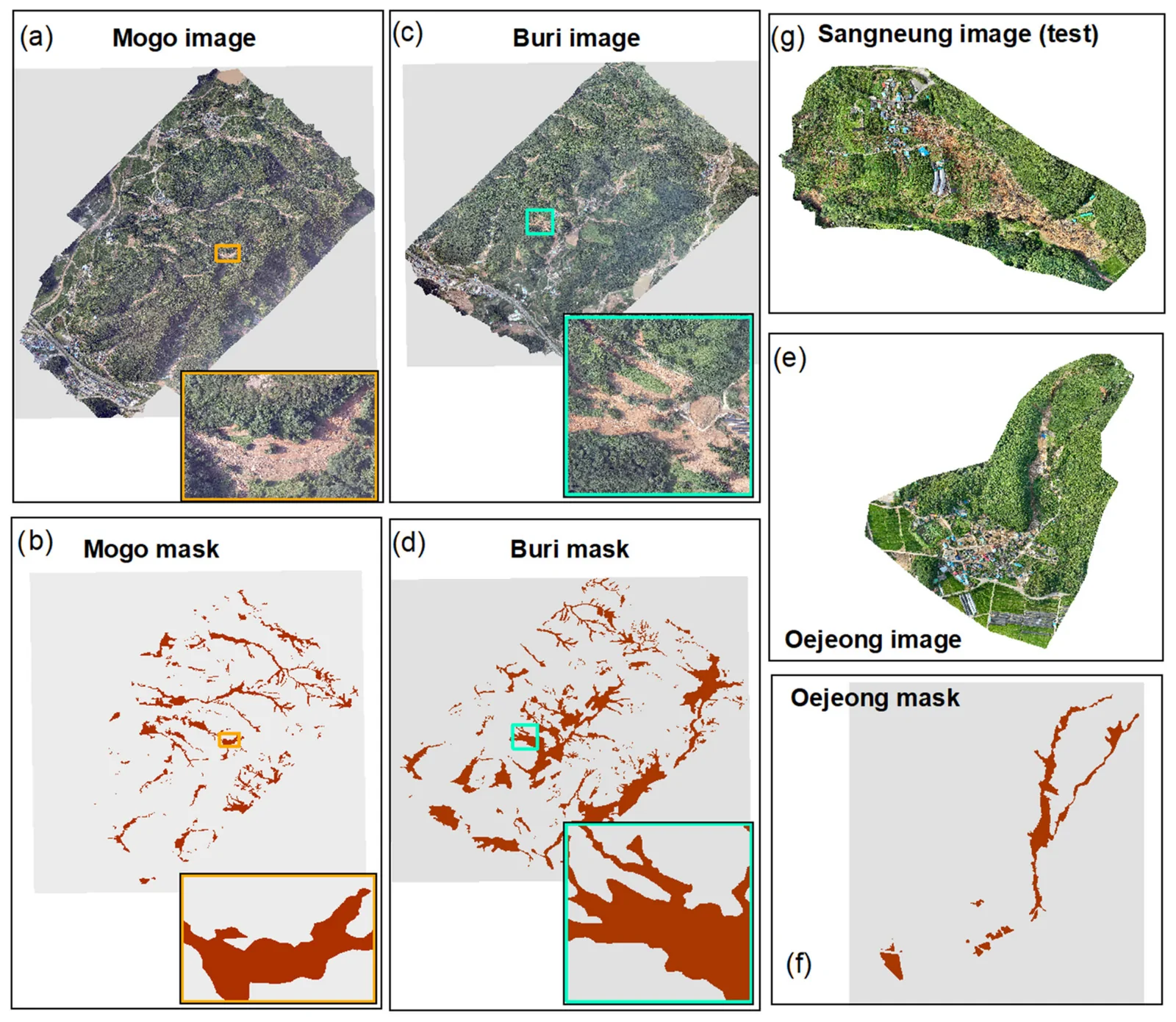
UAV data and landslide mask; (**a**) covering Mogo village with a zoom-in in the golden yellow rectangle, (**c**) covering the Buri village with a zoom-in in the turquoise rectangle, (**b**,**d**) corresponding ground truth masks (in terracotta), (**e**,**f**) are UAV image and ground truth mask (in terracotta) of the Oejeong village, and (**g**) UAV image covering the Sangneung village.

The four landslide sites are projected to be captured by the DJI Matrice 350 RTK UAV, with the Zenmuse L2 sensors, manufactured by DJI Technology Co., Ltd., Shenzhen, China, after the landslide event. The flights were conducted at an altitude of 110 m with an overlap of 80% in the longitudinal and 70% in the lateral direction; thus, we acquired images with a resolution of 3.85 cm after terrain collection. The images are mosaicked and projected in the WGS 1984, UTM, Zone 52N coordinate system for each site, as indicated in Figure 2. The LS boundaries are digitized in rooms (shapefiles) based on the information recorded in the field and expert knowledge. The LS boundary shapefiles are converted to grid data with the same resolution as the images for training dataset creation. The Sangneung village is chosen for the testing area, and a ground-truth mask is vectorized for evaluating the testing result. The images and masks of the Mogo, Buri, and Oejeong are patchified into 512 × 512 patches with zero percentage of overlap, resulting in 13,411 images and the same number of corresponding masks. The training dataset (training and validation) is built from 70% and 30% of the total images and masks, which were randomly split.

### 4.2. General Study Framework

**Figure 3:**
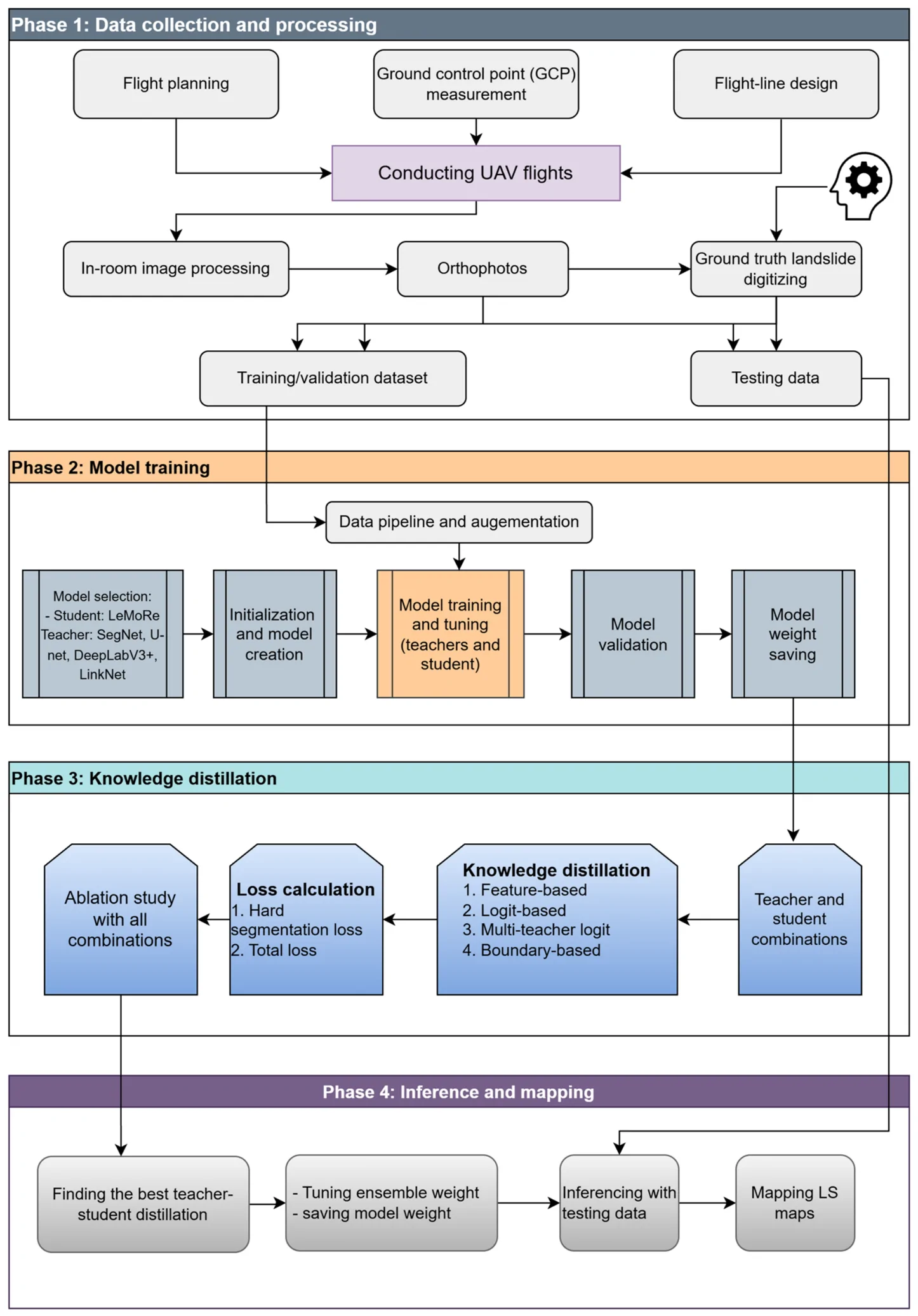
Scheme framework of deep learning knowledge distillation for UAV data pipeline.

To achieve the study goals, the study framework is designed (Figure 3), consisting of four phases: UAV data imaging and processing; training and tuning deep learning models; knowledge distillation (KD) and ablation study; and, in the last phase, using the best model for inference and LS mapping. The UAV data scanning is described in detail in Section 4.1. In the deep learning model training phase, based on a literature review, we select LeMoRe, a newly developed, lightweight model, as the student, and SegNet, U-Net, DeepLabV3+, and LinkNet as the teacher models. By choosing such models, we can leverage the advantages of models that have been validated and tested through numerous previous studies and utilize the knowledge that the models have learned from standard datasets like ImageNet. All the models are trained and tuned with the same data pipeline, configurations, and sources as in Table 1. In the KD phase, student–teacher combinations are established, and then the KD process is performed with four distilled features and two losses. The ablation study is implemented to figure out which ensemble is the best for the next step. The best model weights are saved to a local disk for inference with the testing data. Note that the testing orthophoto is a georeferenced image; thus, after the patch splitting and mask segmentation, the predicted masks must be merged and transferred to the original projection. Hence, the final merged mask is georeferenced and can be overlaid with other GIS layers.

### 4.3. Student Architecture Description

**Figure 4:**
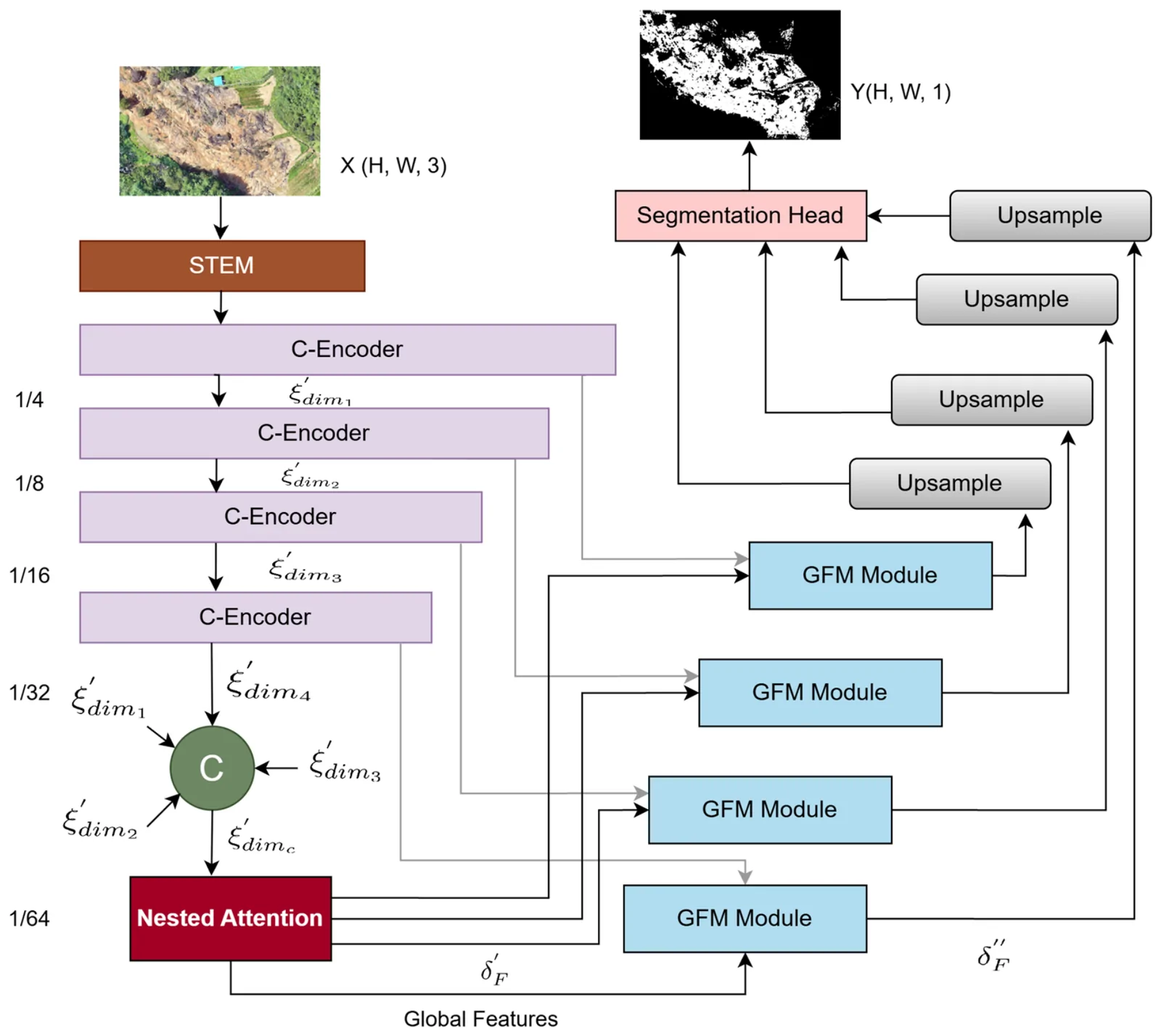
LeMoRe architecture, adapted from [27].

LeMoRe is chosen for the learner model as it is benchmarked as lightweight and has the ability to learn more detailed information for semantic segmentation; the model architecture is depicted in Figure 4 with the downsampling branch including the Cartesian encoder with the nested attention and upsampling branch with GFM modules [27]. Additionally, it is considered not only a pure-light architecture but also a combination of explicit feature modeling through the Cartesian encoder and implicit feature modeling via the nested attention (Figure 4). This design is suitable for LS segments that have irregular shapes, diverse textures, and complex backgrounds such as open ground, exposed rocks, roads, shadows, and vegetation. The network is verified with an effective balance between robustness and efficiency on several datasets, such as ADE20K, CityScapes, Pascal Context, and COCO-Stuff. The general working process of LeMoRe is formulated as [27]:

(1) $$\begin{array}{l} I_{t},I_{f},I_{l}=\rho_{t}(I),\rho_{f}(I),\rho_{l}(I)\\ \xi_{\dim}=\zeta_{t}^{1\times 1}\left(I_{t}\right)+\zeta_{f}^{1\times 1}\left(I_{f}\right)+\zeta_{l}^{1\times 1}\left(I_{l}\right) \end{array}$$

where, *t*, *f*, and *l* are generating Transverse, Frontal, and Lateral views, respectively. The spatial permutation along the direction *x* in [*t*, *f*, *l*] is presented as *ρ_x_*(*I*).

### 4.4. Teacher Model Description

#### 4.4.1. SegNet

SegNet is a fully convolutional neural network (CNN) architecture for semantic pixel-wise segmentation, which was introduced by Badrinarayanan et al. (2017) [60]. The network has the characteristic that the input to the upsampling consists of lower-resolution feature maps, and the decoder employs pooling indices from the corresponding encoder’s max-pooling step to perform non-linear upsampling. Thus, the need for upsampling in learning is eliminated. Furthermore, dense feature maps are generated by the trainable filters in the sparse upsampled maps. Compared to popular architectures such as FCN, DeepLab-LargeFOV, and DeconvNet, it is claimed to have comparable accuracy, memory usage, and computational time of inference [60].

#### 4.4.2. U-Net_(resnet34)_

U-Net is a widely applied semantic segmentation model that was initially developed for biomedical image segmentation, which was proposed by Ronneberger et al. (2015) [24]. The U-Net architecture has the expressiveness of downsampling and upsampling branches in a U-shape, which was hence termed U-Net. The model has become well-known for its simplicity and robustness in object segmentation. The feature maps are downscaled and upscaled through the encoder and decoder effectively by using a transposed convolution. The network uses skip connections that concatenate the high-resolution features from the encoder to the corresponding decoder [24]. In addition, the pretrained backbone of ResNet34, available at ImageNet, is integrated into the network to leverage the trained knowledge from the ImageNet dataset.

#### 4.4.3. DeepLabv3+_(resnet50)_

The larger-size network of DeepLabV3+ by Chen et al. (2018) [21] is a semantic segmentation model developed from the previous model of DeepLabV3 in the DeepLab family. The model has the full capacity of the formal version with the innovation of an integrated encoder–decoder structure to support refining segmented boundaries and recovering finer details that are not found in the previous versions. What is more, the strengths of atrous convolution for context and skipped connections are employed by utilizing a hybrid method of leveraging the Atrous Spatial Pyramid Pooling (ASPP) from the previous versions and by concatenating with the low-level feature maps from the backbone. Thus, the boundary extraction is determined effectively [21]. Similarly, the ResNet50 backbone, a large network in the ResNet family, is integrated into DeepLabV3+.

#### 4.4.4. LinkNet_(resnet50)_

In 2017, Chaurasia and Culurciello proposed a pixel-wise semantic segmentation model, named LinkNet, not only for visualizing scene understanding but also for efficiency and aiming for uses in real-time applications, as it is light and fast [26]. LinkNet is designed similarly to the U-Net architecture; thus, the computational complexity is optimized. However, while the U-Net utilizes skip connections, the encoder’s feature maps are embedded and input into the decoder’s upsamples in LinkNet. Therefore, it calls for more spatial preservation and memory efficiency. The pretrained ResNet backbone is also embedded in LinkNet.

### 4.5. Multi-Teacher–Student Knowledge Distillation

This is the central part of this study methodology, where LeMoRe distills knowledge from multi-teacher models to learn, thus termed “LeMoRe-MTKD”. In the distillation process, each teacher’s logits undergo temperature scaling and are converted into probabilities; these probability maps are then combined via weighted averaging. Finally, the fused probability is converted back into logits for use in logit KD and boundary KD. Four aspects of the feature-based, single-teacher (and multi-teacher) logit, and boundary-based distillation are implemented as follows;

- The feature-based distillation

First, the student and teacher return both logits (L) and features (F):

(2) $$L_{\mathrm{feat}}=\;{\left\|\frac{F_{s}}{\left\|F_{S}\right\|}-\;\frac{A\left(F_{t}\right)}{\left\|A\left(F_{t}\right)\right\|}\right\|}_{2}^{2}$$

where *F_s_* represents the student’s bottleneck features, and *F_t_* represents the teacher’s bottleneck features. The *A*(•) is the 1 × 1 channel adapter. As the teacher’s features are detached, the gradient is not passed back to the teacher. Therefore, students learn not only the final outcome but also how the teacher represents features at a deeper level. This is why feature distillation is suitable for single-teacher distillation, as both have relatively compatible CNN-like feature maps.

- The single-teacher logit distillation

*L_logit_* = *T*^2^*D_KL_*((*p_t_*‖*p_s_*) (3)

$$z_{s}^{,}=\mathrm{concat}\left(-z_{s},\;z_{s}\right),\;z_{t}^{,}=\mathrm{concat}(-z_{t},\;z_{t})$$

$$p_{t}=\mathrm{softmax}\left(\frac{z_{s}^{,}}{T}\right),\;p_{s}=\mathrm{softmax}\left(\frac{z_{t}^{,}}{T}\right)$$

where the student’s logit and teacher’s logit and softmax temperature are *z_s_* and *z_t_*, *p_s_* and *p_t_*, respectively. T and *D_KL_* are the temperature and Kullback–Leibler (KL) divergence. *T*^2^ is the gradient correction factor, and it is commonly used in KD.

- The multi-teacher logit distillation

Each teacher produces one logit map that is divided by the temperature (*T*) and converted to probability maps by the sigmoid function.

$$p_{k}=\sigma \left(\frac{z_{k}}{T}\right),\;p_{\mathrm{com}}=\mathrm{clip}\left(\sum_{k=1}^{K}w_{k}p_{k},\epsilon ,\;1-\epsilon \right)$$

(4) $$z_{\mathrm{com}}=\mathrm{TLog}\left(\frac{p_{\mathrm{com}}}{1-p_{\mathrm{com}}}\right),\;w_{k}\geq 0,\;\sum_{k=1}^{K}w_{k}=1$$

where *z_k_* is the output logit map of teacher number, and *σ*(∗) is the sigmoid function. *T* is the temperature used for all teachers. *w_k_*, *p_com_*, *z_com_* are the standardized contribution weights of teacher k, the probability map after weighted averaging and clipping, and the logit map reconstructed from the joint probability, respectively. ∈ is a small number to prevent infinity in the logit and is set to 10^−4^.

Hence, the student does not learn from an individual teacher but from the ensemble soft target as

(5) $$L_{\mathrm{logit}}^{\mathrm{multi}}=T^{2}D_{\mathrm{KL}}\left(\left.\mathrm{softmax}\left(\frac{{\overset{´}{z}}_{\mathrm{com}}}{T}\right)\right\|\mathrm{softmax}\left(\frac{{\overset{´}{z}}_{s}}{T}\right)\right)$$

$$z_{\mathrm{com}}^{\prime }=\mathrm{concat}\left(-z_{\mathrm{com}},\;z_{\mathrm{com}}\right),\;z_{s}^{\prime }=\mathrm{concat}(-z_{s},\;z_{s})$$

where, *z_s_* is the student logit map. This is the most important part of the contribution of the multi-teacher logit distillation.

- The boundary-based distillation

It is useful for landslide segmentation as the boundary is focused, which is expressed by

(6) $$L_{\mathrm{boundary}}^{\mathrm{multi}}=\sum_{k=1}^{K}w_{k}{\left\|B\left(P_{s}\right)-\left.B\left(P_{\mathrm{tk}}\right)\right\|\right.}_{1}$$

$$B\left(P\right)=\sqrt{G_{x}^{2}+G_{y}^{2}+\in },\;G_{y}=S_{y}*P\mathrm{and}G_{x}=S_{x}*P,\;P=\sigma (z)$$

where *P* is the probability of the foreground and the gradient (*G*) is computed for the *x* and *y* directions by the Sobel filters (*S_x_*_,_*S_y_*). The gradient magnitude is calculated as the *G* for the directions *x* and *y.* The ∈ is a small number. The goal of this distillation is to help students preserve the shape and boundaries of the landslide area rather than simply optimizing the number of foreground/background pixels.

### 4.6. Model Evaluation

To evaluate the deep learning model performance, we apply the standard metrics of recall (Rc), precision (pre), F-score (F1), accuracy (ac), and intersection over union (IoU). The F1 score is calculated from the TP, FP, and FN values for all images; then, it is summed to obtain a single F1 score for the entire evaluation set. As these metrics are presented frequently in the previous literature, such as [61,62], we skip presenting them in detail. For monitoring the training process, the losses are indicated by Equations (6)–(9) as follows:

✓ The hard segmentation distillation loss

The hard segmentation loss includes the BCE and the Dice, which are defined by Equations (7) and (8).

*L_seg_* = 0.5*L_BCE_* + 0.5*L_DICE_* (7)

(8) $$L_{\mathrm{DICE}}=1-\frac{2\sum_{i}p_{i}*y_{i}+\in }{\sum_{i}p_{i}+\sum_{i}y_{i}+\in }$$

The student model still learns directly from the ground-truth masks. This is important because the teacher can make incorrect predictions. Hard loss prevents students from blindly copying the teacher’s mistakes.

✓ The total distillation loss

*L_total_* = *λ_h_L_seg_* + *λ_l_L_logit_* + *λ_f_L_feat_* + *λ_b_L_boundary_* (9)

(10) $$L_{\mathrm{total}}^{\mathrm{multi}}=\lambda_{h}L_{\mathrm{seg}}+\lambda_{l}L_{\mathrm{logit}}^{\mathrm{multi}}+\lambda_{b}L_{\mathrm{boundary}}^{\mathrm{multi}}$$

where lambda hard (*λ_h_*) = 1.0, *λ_l_* = 1.0, *λ_f_* = 0.5, and *λ_b_* = 0.25.

### 4.7. Ablation Study

As each teacher model contributes to the learner model differently, we designed the ablation study using subsets of combinations involving one, two, or three teachers. The student (LeMoRe) is also trained as a plain model for comparison with the subsets (Table 2). All the ablation model runs are implemented with the same computational and configuration settings as described in Table 1 for a fair comparison and for finding the best combination. Although there are a dozen evaluation metrics, for the purpose of semantic segmentation, the IoU and F1 are used for evaluation in this task. In addition, to test the contributions of each KD component (feature-based, logit, feature, and boundary), ablated implementations are conducted for the ST-KD and KD-full.

## 5. Results

### 5.1. Knowledge Distillation Implementation

#### 5.1.1. Monitoring Losses

**Figure 5:**
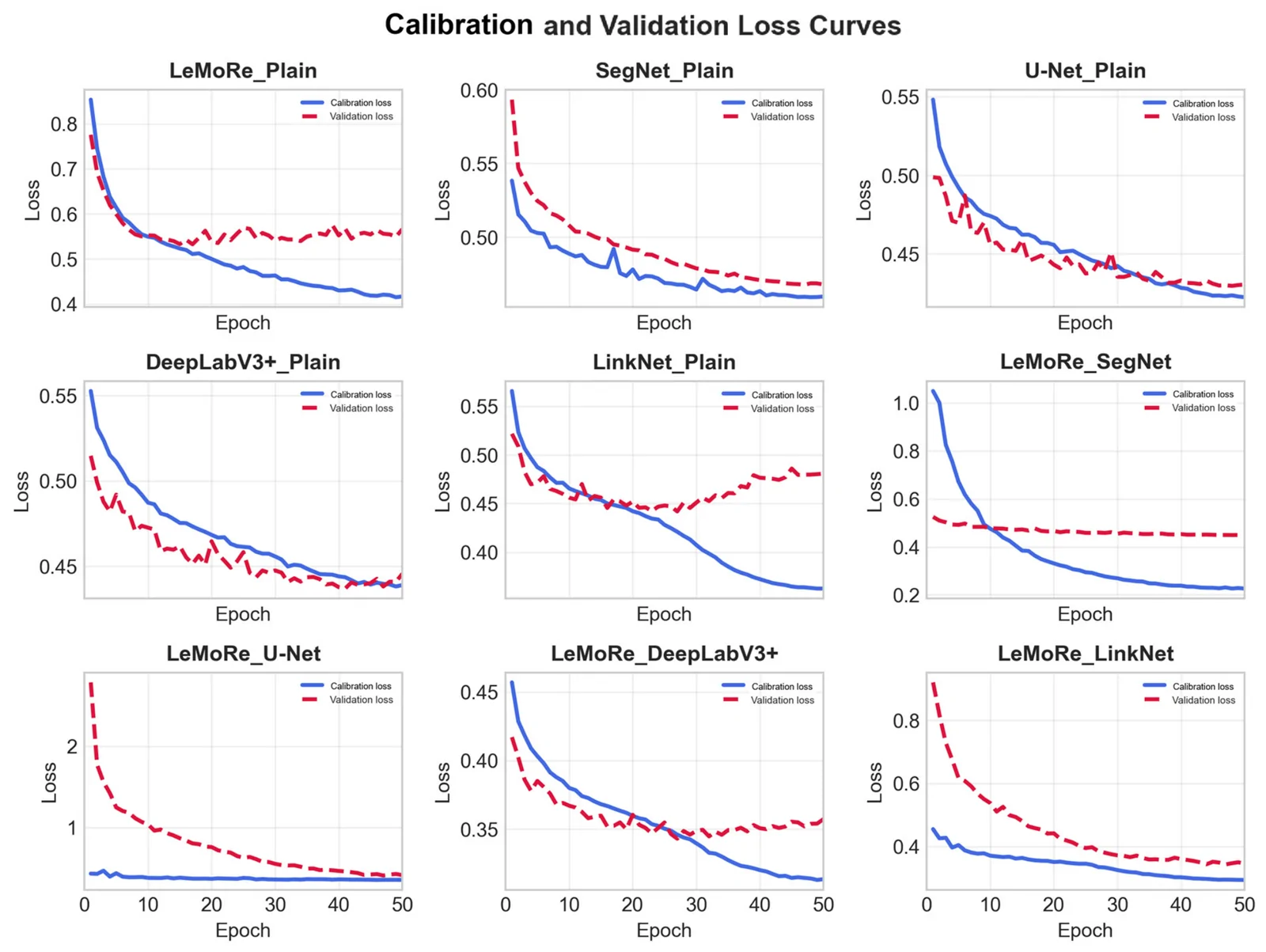
Student and teacher calibration and validation total loss of plain and in-pair KD (single KD).

There were variations in trend and values of the calibration losses (broken red lines) between the learner (LeMoRe), teachers, and the single student–teacher pairs when compared with the validation losses (continuous blue lines) (Figure 5). However, the common trend was of decreased losses in both the calibration and validation phases except for the validation of the LinkNet_plain from the 30th to the 50th epoch. This trend shows the training process was going well. As shown in the figure, with knowledge distillation (KD), the losses were smoother than the greater fluctuations observed in the single original models, particularly for U-Net and DeepLabV3+. In addition, referring to the number of training and validation epochs, the training and validation losses showed that SegNet, U-Net, KD LeMoRe_U-Net, LeMoRe_SegNet, and LeMoRe_LinkNet can decline further, or the models can be improved. However, the validation losses of LeMoRe_plain, LinkNet_plain, DeepLabV3+_plain, and LeMoRe_DeepLabV3+ increased at the final epochs, meaning that the models may be saturated with the training dataset and no further training epochs are needed.

#### 5.1.2. Accuracy Metric Assessment

Table 3 presents the mean accuracy metrics of the validation for the 50-epoch implementation, which compares the plain model (no KD) and the single KD in pairs and the multi-teacher KD of all teachers for LeMoRe with equal weight (0.333) in the last row of Table 3. SegNet achieved the lowest values for most metrics and also achieved lower values with KD mode. On the other hand, LinkNet performed the best with both plain and KD mode with recall, precision, and F1 greater than 0.8 and IoU of 0.74. LeMoRe-U-Net and the MTKD with equal weight have the same IoU (0.653). However, other indicators for LeMoRe-MTKD (equal weight) are slightly better. It was a surprising result that DeepLabV3+ with pretrained ResNet50 (larger than ResNet34) did not surpass the lighter network of U-Net (ResNet34). Additionally, at this stage, we plotted the predicted LS masks and overlaid them with the ground-truth (GT) masks for comparison (TP, TN, FP, and FN), as shown in Appendix A. Although the LeMoRe-LinkNet KD proved high performance, the multi-teacher KD still provided additional improvement, meaning that other teachers can provide additional information via their output probability distributions. However, the complementarity among teachers is considered a reasonable explanation, though it is not a mechanism that has been confirmed through direct error analysis.

#### 5.1.3. Performance Efficiency Assessment

The deep learning model’s efficiency is determined by measuring the computational and memory requirements in relation to the model complexity (parameter number), as shown in Table 4 for the student, teachers, and the single- and multiple-teacher KD. In this phase, we applied an identical inference batch size of 1, numerical precision of FP32, and 20 warm-up iterations for fair model efficiency comparisons. The inference time is recorded by averaging the time of 100 runs. CUDA synchronization is performed immediately before and after each run to ensure timing accuracy; meanwhile, the peak GPU memory is also recorded. In the results, LeMoRe indicated the lightest architecture with 3.117 million parameters and remained unchanged when it is trained by the teacher models. On the other hand, LinkNet(ResNet50) is the largest network with over 31.1 million parameters but at the second-highest operational rate of 43.142 Gflops. SegNet was executed at a very high operational rate of 178.216 Gflops, and its latency was 4.529 milliseconds. LeMoRe was the fastest model, processing around 630 frames per second, and its latency was 1.585 milliseconds (the best). In terms of memory consumption, U-Net requires the lowest memory (608.229 MB) in the inference phase, which is in contrast to SegNet with over 1 GB. Overall, LeMoRe and its KD variants have substantially fewer parameters and lower latency than the teacher networks. However, their inference memory consumption is not lower than that of all teacher models. Importantly, knowledge distillation improves the segmentation performance of LeMoRe without increasing its parameter count or computational complexity at inference time.

### 5.2. Ablation Studies

#### 5.2.1. Ablation Component

**Figure 6:**
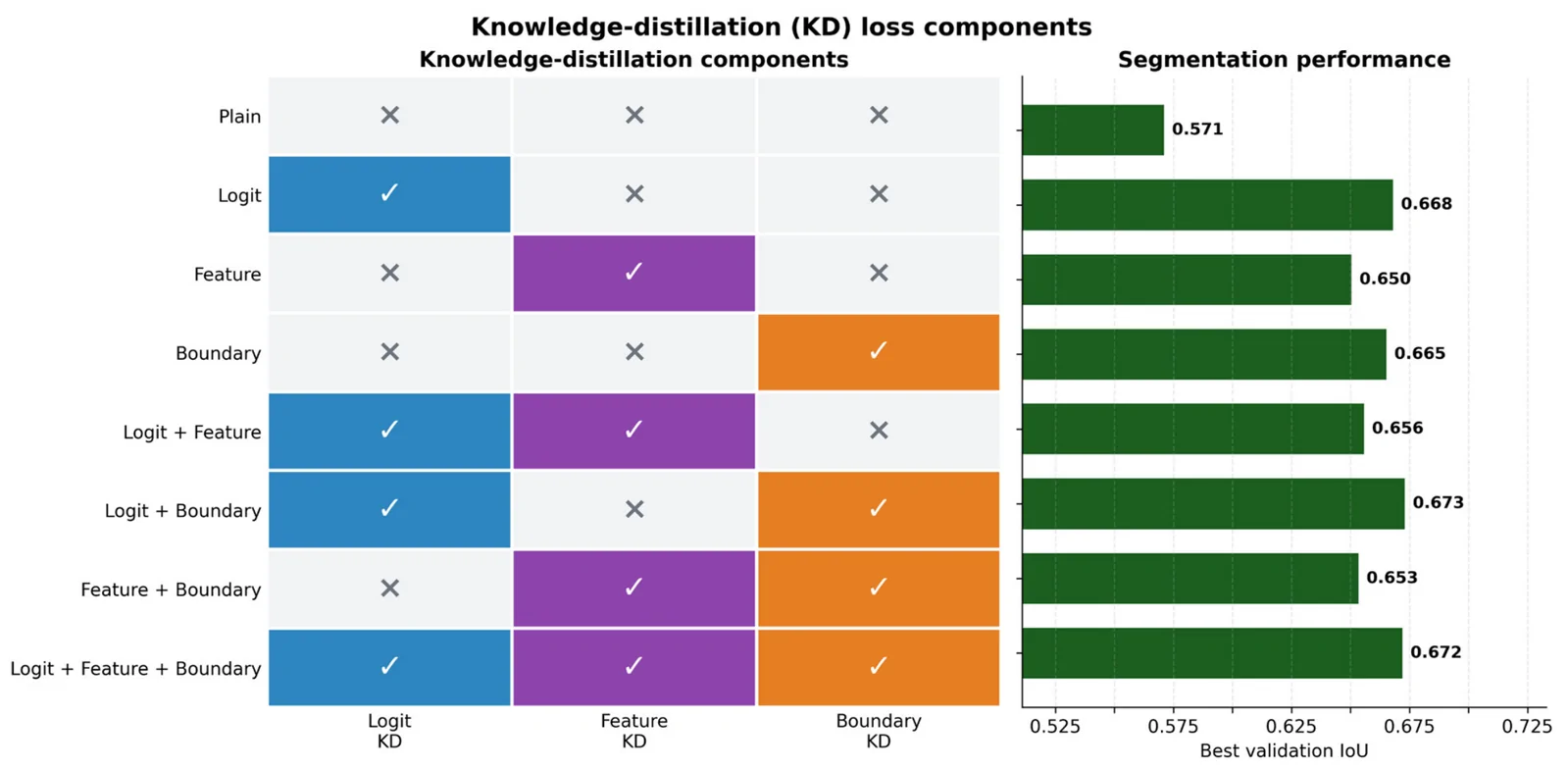
Testing for the effects of the knowledge distillation component on the student network (LeMoRe = Plain); the white tick is used, and x is unused.

The results of measuring the contributions of KD components of the hard loss, logit, feature, and boundary to the plain LeMoRe are shown in Figure 6, showing that the standalone LeMoRe with the hard loss achieved the lowest IoU (0.571) in its validation, while the logit contributed significantly (0.668). In contrast, the KD feature added to the IoU of KD-LeMoRe by approximately 0.079. However, the KD boundary made a positive contribution again, resulting in an increase in IoU to 0.665. Although the components complement each other, resulting in increased accuracy when all components are distilled (IoU = 0.672), the Logit+boundary combination still achieves the best results (IoU of 0.673) and is utilized in the subsequent step to attain the method’s peak accuracy.

#### 5.2.2. Multi-Teacher Knowledge Distillation (MTKD)

The validation accuracy metrics of all the LeMoRe variants are tabulated in Table 5, ranging in rows from single-teacher to multiple-teacher equal-weighted KD. The subset of LeMoRe ← [U-Net, DeepLabV3+, LinkNet] achieved the best recall of 0.876, F1 of 0.875, and IoU of 0.778. The contribution of SegNet was minimal with lower metrics where it appears; thus, its omission from the best MTKD combination was an appropriate reason. Across the comparison between two-teacher and three-teacher KD, there were slight improvements in the three-teacher KD performances. Furthermore, when it comes to semantic segmentation, the IoU is especially important; therefore, although LeMoRe ← [U-Net, DeepLabV3+, LinkNet] does not completely prevail across all metrics, it is still the best choice to include in the next step. At this stage, the maps of validated segments were plotted in Appendix B, where the predicted results of the plain LeMore and the best model subset are comparable.

### 5.3. Weight Tuning of the Best Subset

**Figure 7:**
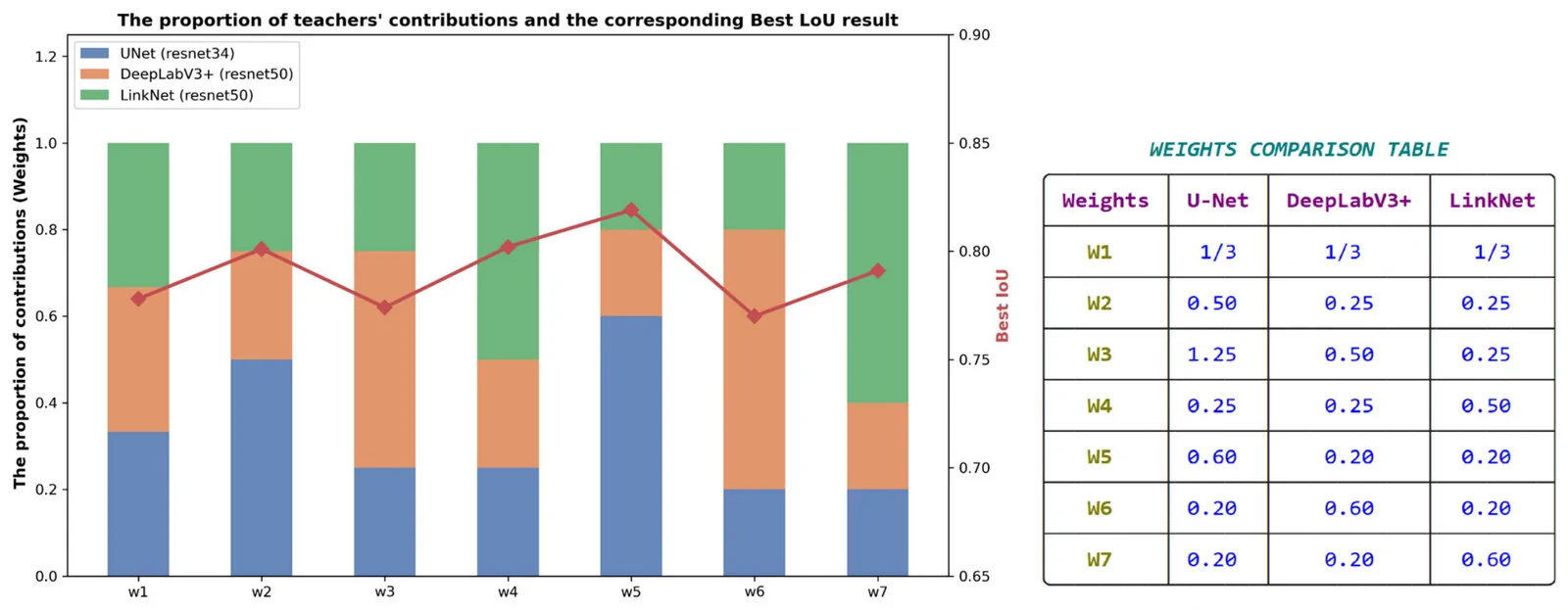
Results of the weight proportions of the teacher’s contribution to the IoU in the model validation phase.

The best subset is LeMoRe ← [U-Net, DeepLabV3+, LinkNet], which is determined by the ablation study for the MTKD implementation from the previous step. However, to determine the optimal weight proportions for each component model in this combination, the results are presented in Figure 7 from the weight-tuning implementation with seven variants (W1–7). The most precise subset, based on the IoU, was W5 (60% of U-Net/20% of DeepLabV3+, and/20% of LinkNet) of IoU 0.819, meaning that this subset is leaning on U-Net. Nonetheless, W6 had the lowest IoU (around 0.770). Further information on the comparisons between the seven variants’ predicted masks and the ground truth (GT) masks in their validations is plotted and provided in Appendix C.

### 5.4. Landslide Mapping

**Figure 8:**
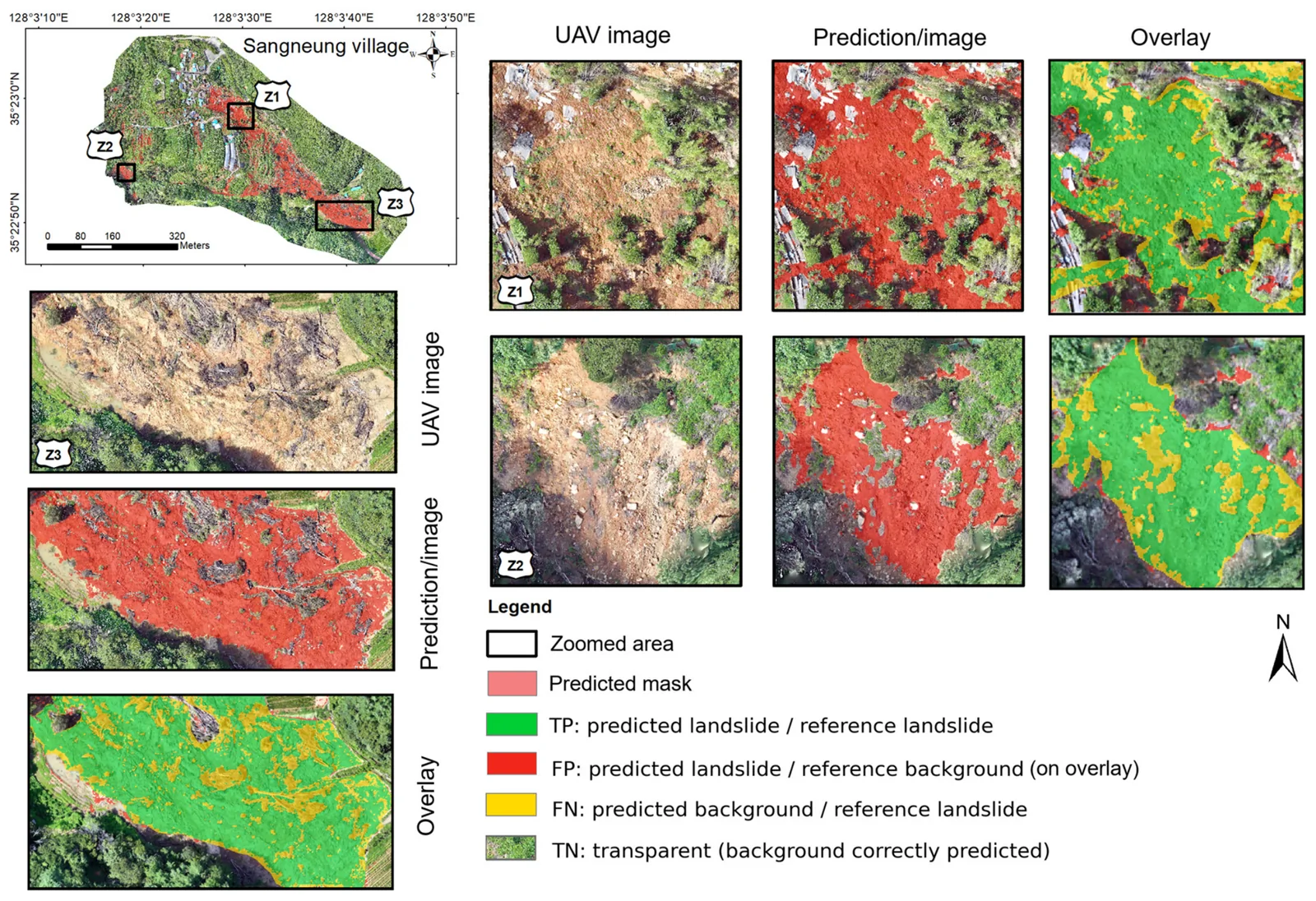
Landslide maps by multi-teacher knowledge distillation of LeMoRe, LeMoRe ← [U-Net, DeepLabV3+, LinkNet] with W5 (60% U-Net, 20% DeepLabV3+, 20% LinkNet), in Sangneung village.

The inference results for the testing site of Sangneung village by the best MTKD subset applied with the W5 weight configuration are presented in Figure 8. Three zones (Z1–3) are selected for larger visualization, compared to the original UAV scenes, predicted mask, and overlaid all layers. Overall, the predicted mask highlighted most landslide-damaged areas (in red) correctly in the main map (top-left). However, when looking at more detail, zooming in on Z1, some disposed stones in the upper-right corner could be predicted as LS-affected areas. Additionally, there were some tree-shaded areas that could be landslide areas, but these were misclassified as LS (false positives). The similar tree-shaded areas in the bottom-right corner in Z2 were predicted as non-LS; however, even by human-eye observation, it is difficult to recognize whether they are false positives or true negatives. Aside from those doubts, the model has very accurately identified the areas affected by landslides in lime green on the overlay maps. The larger downstream area (Z3) was heavily affected by the LS. By zooming in, most LS-affected areas were masked correctly, and the dead-lying trees were predicted as non-LS. In the inference implementation, the entire Sangneung village image was patchified into numerous patches and merged back to the original size; however, the masks were combined seamlessly with no artifacts on the borders. Quantitatively, the agreement between ground truth and predicted masks of Sangneung is shown by an IoU of 0.678, F1-score of 0.808, and recall of 0.725 calculated from a confusion matrix.

## 6. Discussion

### 6.1. Knowledge Distillation Effectiveness

In addition to model accuracy, their effectiveness is crucial, especially for rapid implementation tasks under computational constraints [63]. In this study, LeMoRe and its knowledge distillation (KD) variants have been shown to be highly efficient with only 3.1 million parameters and 26.5 GFLOPs; in particular, the number of parameters does not increase after KD while still leveraging advanced knowledge from the teacher models. Under resource limitations, lightweight and effective networks will strongly promote applications not only in research but also in practical applications. The improvement of segmentation accuracy and computational cost aspects of KD has been proven and discussed in recent studies, such as Liu et al. (2026) [64], and reviewed in Silva et al. [65]. In addition, at the larger national scale in China, the KD effectiveness is reaffirmed in the field of climate studies under physical constraints; nonetheless, the lightweight student model achieved improved model speed, lower memory consumption, and more accurate predictions [66].

### 6.2. Trade-Offs Between Accuracy and Efficiency, and Necessary Ablation Studies

Research efforts to improve deep learning model accuracy often involve developing larger and more complex networks. However, adding network components or combining multiple CNNs generally increases model complexity and computational requirements [67], although such approaches may improve the ability to learn information from training data [68]. Therefore, a trade-off between model accuracy and computational efficiency should be considered. Similar considerations have been discussed in other application fields. Ziran et al. [69] evaluated predictive models by jointly considering accuracy, memory requirements, and computational energy, highlighting the importance of computational requirements in addition to predictive performance.

In this study, knowledge distillation enables the lightweight LeMoRe model to improve segmentation performance without increasing its inference-time model complexity. However, not all KD components contribute equally to student performance. For example, feature-based KD did not improve the segmentation performance in this study and was therefore excluded from the multi-teacher KD. Thus, ablation testing is important for identifying effective KD components. Furthermore, as individual teachers may contribute differently to the student model, teacher weight tuning is necessary to determine appropriate contribution ratios. In this study, U-Net was assigned the largest weight in the best-performing teacher-weight configuration. In addition, although LeMoRe achieved a higher IoU after distillation compared to individual teachers, this result does not imply that the student consistently outperforms the teachers but rather reflects the combined effects of hard-label supervision, soft targets from multiple teachers, and regularization mechanisms during training.

### 6.3. Knowledge Distillation Limitations

Although the DL framework has advantages as mentioned, it has a few limitations depending on the teachers during the training and validation process; particularly when an error is raised from any model, the entire process needs to be re-implemented. That takes more time than training individual student and teacher models. Thus, the training and validation costs are increased dramatically [70,71]. Otherwise, when the training is completed, the distilled student is as light as the original one (LeMoRe). Secondly, the size of the student network is considerably smaller than that of the teacher networks; therefore, it cannot fully reproduce the representation, multi-scale context, and segmentation capabilities of the ensemble teachers. KD can only transfer the knowledge that the student’s capacity can absorb [72]. Thirdly, in this study, the LeMoRe model learns primarily from output/boundary supervision and has not yet fully exploited intermediate complementary representations (not feature KD) from each teacher. Feature matching between heterogeneous architectures often requires carefully designed adapter/mapping modules. In addition, note that this experiment is based on the random 77/30 data split for the training and validation data; it may result in neighbor patches appearing on both sides and resulting in data leakage. Feasibly, validation data should be used from independent sites. Finally, hyperparameter variants such as changes in learning rate and random seeds could have an influence on the model output and KD method; e.g., training with different random seeds will help to further evaluate the stability of the results. However, for consistent comparison in the entire modeling process, this study intentionally fixed all of them as in Table 1.

### 6.4. Practical Implementations

Once the lightweight LeMoRe is distilled, it can provide improved segmentation performance while maintaining the inference-time model complexity of the original lightweight architecture. Its small parameter count and low latency indicate potential for resource-constrained applications; however, deployment on embedded or edge hardware was not evaluated in this study and requires further validation. Even though there were false positives for tree shadows, the dead trees may be predicted as LS-affected areas. However, this error might not be a methodology issue but rather that the training data were not representative of all LS cases (the lack of tree shadows and dead trees in the training dataset). This problem will be solved with a larger training dataset. On the other hand, other LS details and boundaries are correctly segmented. In addition, with UAV images with very high resolution (in this experiment, 3.8 cm), the lightweight KD LeMoRe is highly practical, as the UAV training data are typically enormous, like the three sites of Mogo, Buri, and Oejeong, which have more than 13 thousand 512 × 512 images and a storage size of 6.7 GB. Hence, for a slower CPU and smaller GPU, it could be a significant challenge to implement with large CNNs. In addition, the UAV imagery contains a larger amount of detailed information; sometimes, it is noise, meaning that the CNNs need to learn more from the data (more images) to recognize the noise. Thus, the dataset increases dramatically in size when the UAV data resolution is growing. That is an inherent conflict. Therefore, choosing an appropriate spatial resolution of the training images is crucial and should be based on the required accuracy, particularly the IoU for the semantic segmentation tasks. Thus, it makes the modeling more practical for real applications.

## 7. Conclusions

This study evaluated a multi-teacher knowledge distillation (KD) framework that transfers knowledge from four teacher models to the lightweight LeMoRe CNN model for landslide semantic segmentation using UAV imagery. The experimental results demonstrated that KD improved the segmentation performance of the student model without increasing its inference-time model complexity. The lightweight LeMoRe architecture (3.117 M parameters) therefore provides a potential approach for processing high-resolution UAV imagery for landslide mapping. Although some misclassifications were visually observed in tree-shadowed areas at the Sangneung testing site, the predicted masks showed good visual correspondence with the apparent landslide-affected areas in the UAV imagery. Overall, the proposed approach has the potential to support post-landslide investigation using UAV imagery, particularly in mountainous and difficult-to-access areas.

## Figures and Tables

**Table 1:** Hyperparameter and hardware/software configuration for deep learning model training and distillation.

Parameter	Type/Values	Parameter	Type/Values	Sources	Hard/Software/Version
Image size	int = 512	Amp	bool = True	CPU	AMD Ryzen 99950X 16-core processor
Seed	int = 42	Threshold	float = 0.5	GPU	NVIDIA RTX 6000 Blackwell
Batch size	int = 16	Early stopping patience	int = 10	Memory	64 GB
Epochs	int = 50	Temperature	float = 4.0	Python	3.11.14
Optimizer	AdamW	Lambda hard	float = 1.0	Pytorch	2.15
Learning rate teacher	float = 1 × 10^−4^	Lambda logit	float = 1.0	CUDA	13.0
Learning rate student	float = 1 × 10^−3^	Lambda feature	float = 0.5	OpenCV	4.7.0
Weight decay	float = 1 × 10^−4^	Lambda boundary	float = 0.25		

**Table 2:** Ablation study design for KD of LeMoRe with single and multi-teachers; ✔ is used and - is unused.

ID	Student/Teacher	SegNet	U-Net (ResNet34)	DeepLabV3+ (ResNet50)	LinkNet (ResNet50)	No. Teachers
B0	LeMoRe	-	-	-	-	0
S1	LeMoRe-ST	✔	-	-	-	1
S2	LeMoRe-ST	-	✔	-	-	1
S3	LeMoRe-ST	-	-	✔	-	1
S4	LeMoRe-ST	-	-	-	✔	1
D1	LeMoRe-2MT	✔	✔	-	-	2
D2	LeMoRe-2MT	✔	-	✔	-	2
D3	LeMoRe-2MT	✔	-	-	✔	2
D4	LeMoRe-2MT	-	✔	✔	-	2
D5	LeMoRe-2MT	-	✔	-	✔	2
D6	LeMoRe-2MT	-	-	✔	✔	2
R1	LeMoRe-3MT	✔	✔	✔	-	3
R2	LeMoRe-3MT	✔	✔	-	✔	3
R3	LeMoRe-3MT	✔	-	✔	✔	3
R4	LeMoRe-3MT	-	✔	✔	✔	3

**Table 3:** Accuracy metrics calculated for the validation phase of plain student and teacher models, single KD, and all-teacher KD with equal weight = 1/3 or ≈0.333; the best metrics are marked in bold.

Model	Accuracy	Recall	Precision	F1	IoU
LeMoRe	0.926	0.754	0.702	0.727	0.571
SegNet	0.921	0.719	0.686	0.702	0.541
U-Net(ResNet34)	0.942	0.814	0.756	0.784	0.645
DeepLabV3+ (ResNet50)	0.933	0.783	0.723	0.752	0.602
LinkNet(ResNet50)	0.956	0.861	0.819	0.839	0.723
LeMoRe-SegNet	0.94	0.712	0.753	0.732	0.577
LeMoRe-U-Net(ResNet34)	0.961	0.776	0.805	0.790	0.653
LeMoRe-DeepLabV3+ (ResNet50)	0.953	0.763	0.751	0.757	0.609
LeMoRe-LinkNet (ResNet50)	**0.968**	**0.873**	**0.828**	**0.850**	**0.739**
LeMoRe-MTKD (equal weight = 1/3)	0.957	0.777	0.804	0.790	0.653

**Table 4:** Metrics of model performance efficiency are recorded in the validation stage; the best metrics are marked in bold.

Model	Parameters (m)	Frames/s (fps)	Gflops	Inference Memory (MB)	Latency (ms/image)	Latency Std (ms)
U-Net (ResNet34)	24.436	237.737	31.399	**608.299**	4.206	0.280
DeepLabV3+ (ResNet50)	26.678	213.740	36.904	641.828	4.679	0.142
LinkNet (ResNet50)	31.178	195.178	43.142	622.781	5.124	0.121
SegNet	16.541	220.791	178.216	1041.063	4.529	**0.020**
LeMoRe-Plain	3.117	629.984	26.546	728.816	1.587	0.039
LeMoRe-STKD	3.117	**630.912**	26.546	728.816	**1.585**	0.043
**LeMoRe-MTKD**	**3.117**	626.899	**26.546**	728.816	1.595	0.041

**Table 5:** Multi-teacher ablation study; ST = single teacher, MT = multi-teacher, KD = knowledge distillation; the best metrics and subset are marked in bold. Note that the U-Net uses ResNet34, DeepLabV3+, and LinkNet with ResNet50.

Model	Training Strategy	Recall	Precision	F1	IoU
LeMoRe ← SegNet	ST-KD	0.712	0.753	0.732	0.577
LeMoRe ← U-Net	ST-KD	0.776	0.805	0.790	0.653
LeMoRe ← DeepLabV3+	ST-KD	0.763	0.751	0.757	0.609
LeMoRe ← LinkNet	ST-KD	0.873	0.828	0.850	0.739
LeMoRe ← [SegNet, U-Net]	MT-KD	0.803	0.818	0.810	0.681
LeMoRe ← [SegNet, DeepLabV3+]	MT-KD	0.821	0.811	0.816	0.689
LeMoRe ← [SegNet, LinkNet]	MT-KD	0.784	0.802	0.793	0.657
LeMoRe ← [U-Net, DeepLabV3+]	MT-KD	0.814	0.815	0.814	0.687
LeMoRe ← [U-Net, LinkNet]	MT-KD	0.792	0.810	0.801	0.668
LeMoRe ← [SegNet, U-Net, DeepLabV3+]	MT-KD	0.808	0.815	0.812	0.683
LeMoRe ← [SegNet, U-Net, LinkNet]	MT-KD	0.773	**0.886**	0.826	0.703
LeMoRe ← [SegNet, DeepLabV3+, LinkNet]	MT-KD	0.808	0.810	0.809	0.679
**LeMoRe ← [U-Net, DeepLabV3+, LinkNet]**	MT-KD	**0.876**	0.874	**0.875**	**0.778**

**Figure A1:**
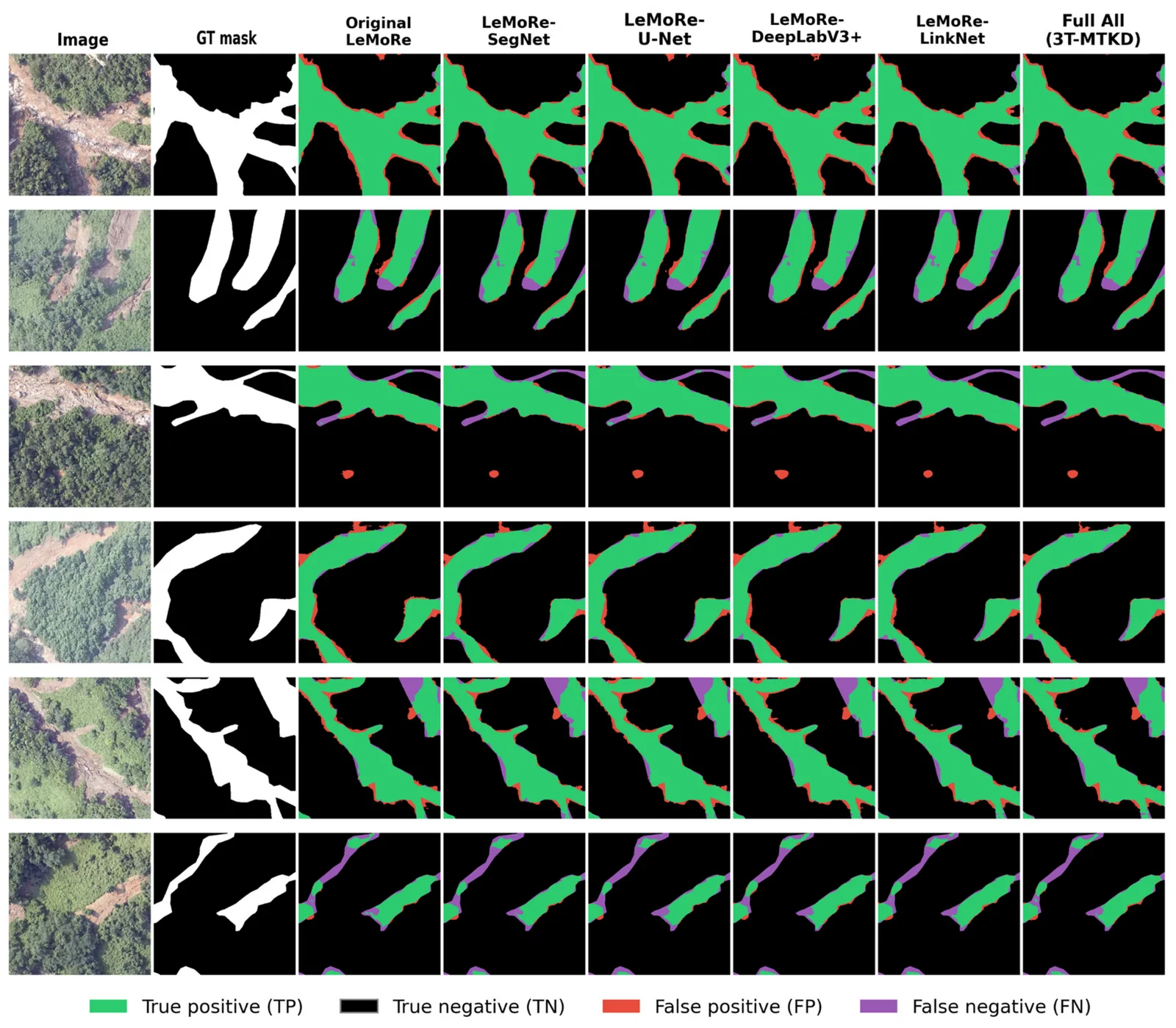
Qualitative Comparison of Original, Single-Teacher, and Multi-Teacher LeMoRe Variants.

**Figure A2:**
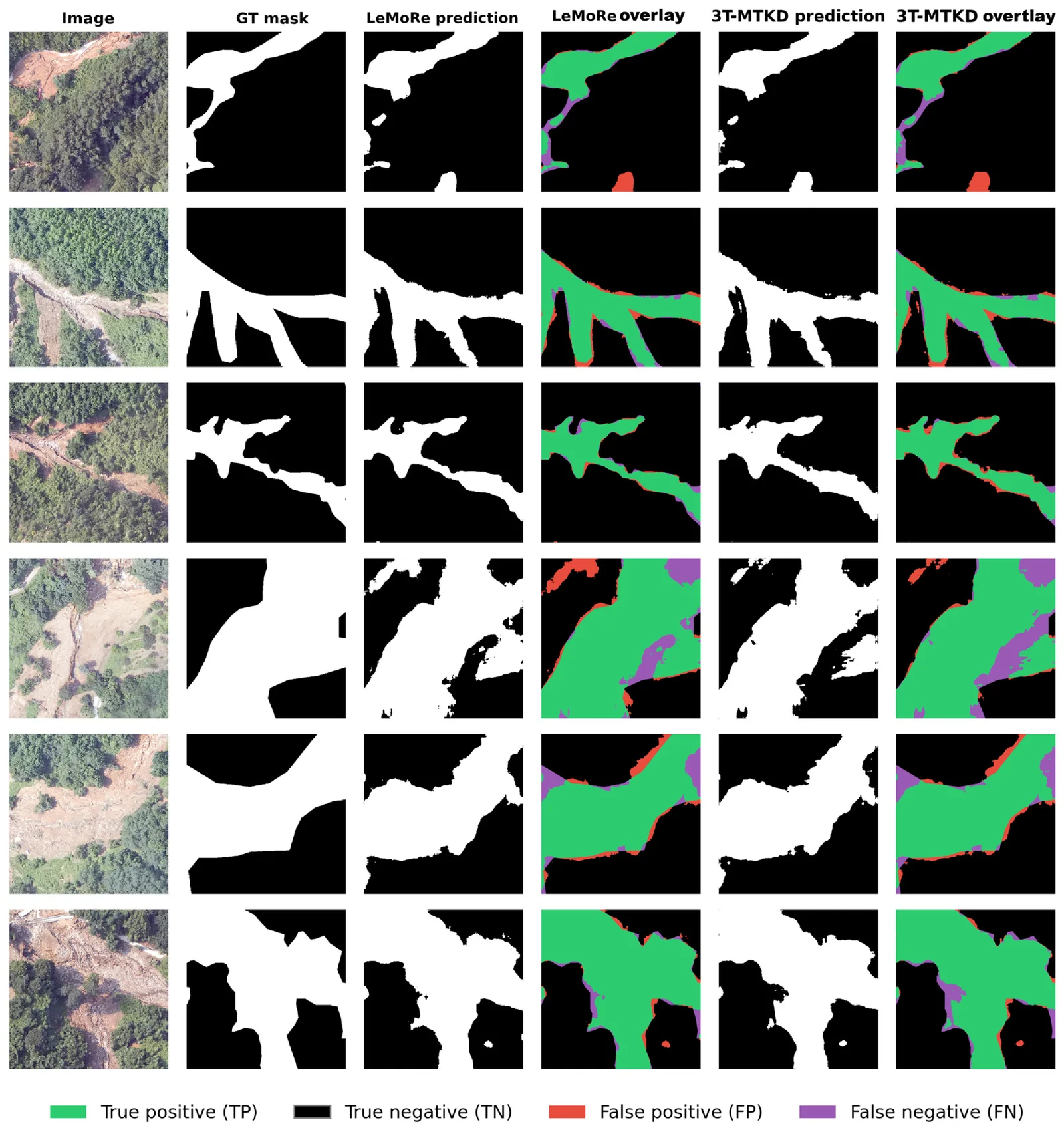
Qualitative Comparison of Original, Single-Teacher, and Multi-Teacher LeMoRe Variants.

**Figure A3:**
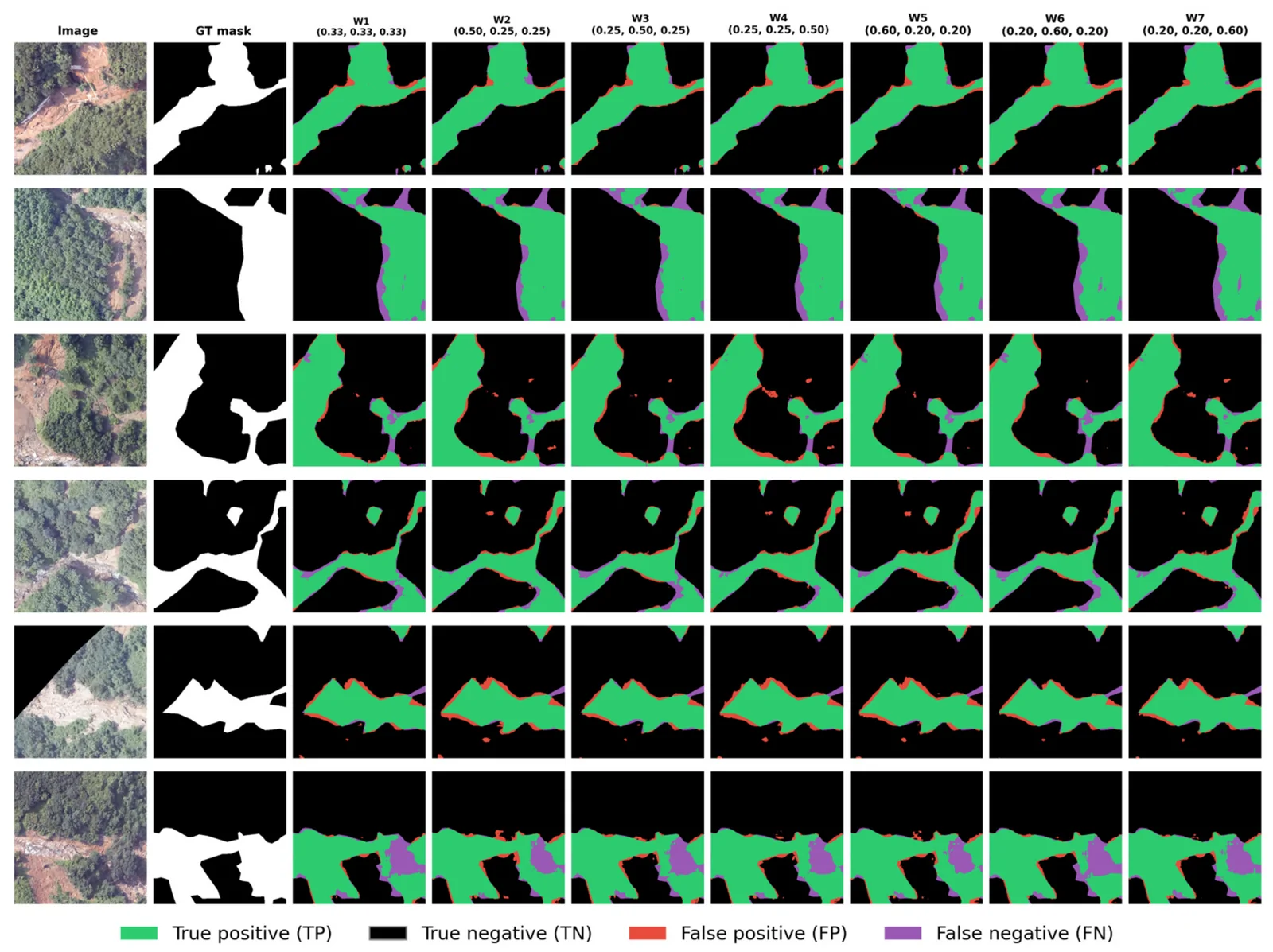
Qualitative Comparison of Phase-4 Three-Teacher Weight Configurations, W1–7 are Weight Proportions.

## Data Availability

UAV image data can be provided upon reasonable request to the corresponding author.
